# Factores asociados a la mortalidad por tuberculosis en Paraguay, 2015-2016

**DOI:** 10.26633/RPSP.2019.102

**Published:** 2019-12-20

**Authors:** Angélica Medina, Lucelly López, Celia Martínez, Sarita Aguirre, Edith Alarcón

**Affiliations:** 1 Programa Nacional de Control de la Tuberculosis Ministerio de Salud Pública y Bienestar Social Asunción Paraguay Programa Nacional de Control de la Tuberculosis, Ministerio de Salud Pública y Bienestar Social, Asunción, Paraguay.; 2 Universidad Pontificia Bolivariana Universidad Pontificia Bolivariana Medellín Colombia Universidad Pontificia Bolivariana, Medellín, Colombia.; 3 Facultad de Ciencias Médicas Universidad Nacional de Asunción Asunción Paraguay Facultad de Ciencias Médicas, Universidad Nacional de Asunción, Asunción, Paraguay.; 4 Organización Panamericana de la Salud/Organización Mundial de la Salud Washington D.C. Estados Unidos de América Organización Panamericana de la Salud/Organización Mundial de la Salud, Washington D.C., Estados Unidos de América.

**Keywords:** Mortalidad, tuberculosis, investigación operativa, Paraguay, Mortality, tuberculosis, operations research, Paraguay, Mortalidade, tuberculose, pesquisa operacional, Paraguai

## Abstract

**Objetivo.:**

Describir las características sociodemográficas y clínico-epidemiológicas y determinar los factores asociados a la mortalidad de personas con diagnóstico de tuberculosis en Paraguay.

**Métodos.:**

Investigación operativa con un diseño de cohortes retrospectivo de los casos diagnosticados con TB en Paraguay entre 2015-2016. Se utilizó la base datos del Programa Nacional de Control de Tuberculosis. Para determinar los factores asociados con mortalidad se utilizaron pruebas chi cuadrado y riesgo relativo (RR) con un intervalo de confianza de 95% (IC95%); además, se ajustó un modelo de regresión múltiple de Poisson robusto. Se utilizó un nivel de significación de 5%.

**Resultados.:**

Se estudiaron 5 141 casos de TB, de los cuales 11,5% fallecieron, los factores que aumentan el riesgo de muerte fueron: sexo masculino (RR: 1,26 IC; 95%: 1,1-1,50), infección con virus de la inmunodeficiencia humana (VIH) (RR: 4,78; IC 95%: 4,04-5,65) y enfermedad pulmonar obstructiva crónica (RR: 1,70; IC 95%: 1,19-2,42). Como factor protector se identificó ser persona privada de la libertad (RR: 0,37 IC 95%: 0,24-0,61).

**Conclusiones.:**

El mayor riesgo de muerte lo presentan los hombres y las personas con coinfección TB/VIH y el menor riesgo, las personas privadas de la libertad. Es necesario mejorar el diagnóstico y seguimiento a los casos de TB, con la efectiva implementación del tratamiento directamente observado (TDO) así como el manejo oportuno de enfermedades asociadas como VIH y enfermedad pulmonar obstructica crónica (EPOC).

## Abstract (en)

**Objective.:**

To describe the socio-demographic and clinical-epidemiological characteristics and to determine the factors associated with the mortality of people diagnosed with tuberculosis (TB) in Paraguay.

**Methods.:**

Operational research with a retrospective cohort design of cases diagnosed with TB in Paraguay between 2015-2016. The database of the National Tuberculosis Control Program was used. Chi-square and relative risk (RR) tests with a 95% confidence interval (95% CI) were used to determine the factors associated with mortality; in addition, a robust Poisson multiple regression model was adjusted. A significance level of 5% was used.

**Results.:**

Five hundred and forty-one cases of TB were studied, of which 11.5% died. The factors increasing the risk of death were male sex (RR 1.26; 95% CI 1.1-1.50), infection with human immunodeficiency virus (RR 4.78; 95% CI 4.04-5.65) and chronic obstructive pulmonary disease (RR 1.70; 95% CI 1.19-2.42). Being deprived of one’s liberty was a protective factor (RR 0.37; 95% CI 0.24-0.61).

**Conclusions.:**

The highest risk of death is presented by men and people with TB/HIV coinfection and the lowest risk is presented by people deprived of liberty. There is a need to improve diagnosis and follow-up of TB cases, with effective implementation of directly observed treatment (DOTS) and timely management of associated diseases such as HIV and chronic obstructive pulmonary disease.

## RESUMO

**Objetivo.:**

Descrever as características sociodemográficas e clínico-epidemiológicas e determinar os fatores associados à mortalidade de pessoas diagnosticadas com tuberculose (TB) no Paraguai.

**Métodos.:**

Pesquisa operacional com desenho de coorte retrospectivo de casos diagnosticados com TB no Paraguai entre 2015-2016. Foi utilizada a base de dados do Programa Nacional de Controle da Tuberculose. Testes de qui-quadrado e risco relativo (RR) com intervalo de confiança de 95% (IC95%) foram utilizados para determinar os fatores associados à mortalidade; além disso, um robusto modelo de regressão múltipla de Poisson foi ajustado. Foi utilizado nível de significância de 5%.

**Resultados.:**

Foram estudados quinhentos e quarenta e um casos de TB, dos quais 11,5% morreram. Os fatores que aumentaram o risco de morte foram sexo masculino (RR 1,26; IC95% 1,1-1,50), infecção por vírus da imunodeficiência humana (RR 4,78; IC95% 4,04-5,65) e doença pulmonar obstrutiva crônica (RR 1,70; 95 % CI 1,19-2,42). Ser privado de liberdade foi um fator protetor (RR 0,37; IC 95% 0,24-0,61).

**Conclusões:**

O maior risco de morte é apresentado por homens e pessoas com co-infecção TB / HIV e o menor risco é apresentado por pessoas privadas de liberdade. É necessário melhorar o diagnóstico e o acompanhamento dos casos de TB, com a implementação efetiva do tratamento diretamente observado (DOTS) e o gerenciamento oportuno de doenças associadas, como o HIV e a doença pulmonar obstrutiva crônica.

La tuberculosis (TB) sigue siendo un problema de salud pública a nivel mundial. La Organización Mundial de la salud (OMS) estimó que en 2017 se presentaron 10,4 millones de casos de tuberculosis (TB), de los cuales fallecieron 1,7 millones. Más de 95% de estas muertes se produjeron en países de bajos y medianos ingresos (1). En el 2016, la tuberculosis estuvo entre las 10 primeras causas de mortalidad en el mundo y entre las cinco principales causas de años de vida potencialmente perdidos (1, 2).

En la Región de las Américas, en 2017 se estimó alrededor de 274 000 casos, con una tasa de 27 por cada 100 000 habitantes, de los cuales fallecieron aproximadamente 7%. La tasa de incidencia estimada de TB en Paraguay fue de 42 por cada 100 000 habitantes, y la mortalidad, de 4,5. Para el 2018, se reportó una tasa de mortalidad para América del Sur de 6,6. Paraguay se encuentra en el cuarto lugar entre los países con mayor tasa, por debajo de Guyana, Bolivia y Perú con tasas por 100 000 habitantes de 23, 12 y 8, respectivamente (1, 3).

Aunque a nivel mundial la tasa de mortalidad está en disminución, aproximadamente 3% al año, y la incidencia es de 2% anual, esto no es suficiente para alcanzar los hitos de la Estrategia Fin a la TB, planificados para el año 2020. Para conseguirlo, las proyecciones muestran que la mortalidad debe disminuir a 4-5% por año y la incidencia, 10% (1, 4). Sin embargo, en la Región de las Américas esto ha sido un poco más lento, ya que la mortalidad ha disminuido 2,5% y la incidencia, 1,6% (3).

En Paraguay, en 2016, se notificaron 2 611 casos de TB todas las formas, de los cuales 2 348 fueron nuevos y recaídas; 90% fueron TB pulmonares y, de estos, 77% tuvieron confirmación bacteriológica. La tasa de incidencia notificada para todas las formas fue 37,2 por 100 000 habitantes. Si bien se observaron avances en la reducción de la mortalidad desde 2005, la tasa quedó estacionada desde 2010 (4,5), presentó una ligera disminución entre los años 2011-2014 (con oscilaciones entre 3,2 y 3,9), un incremento en 2015 a 4,4 y cerró el 2016 con 4,2 por cada 100 000 habitantes.

Se han identificado una serie de factores que predisponen a la muerte por TB: edad, sexo masculino, coinfección TB/VIH, comorbilidades (principalmente diabetes mellitus, DM), resistencia a medicamentos, gravedad de la enfermedad y debilidades en la implementación del tratamiento directamente observado (TDO). Otros factores son la detección tardía, el tratamiento irregular, y el abuso de alcohol y drogas, que acarrean consecuencias fatales (5-7).

En concordancia con lo expuesto, esta investigación tuvo por objetivos describir las características sociodemográficas y clínico-epidemiológicas y determinar los factores asociados a la mortalidad de personas con diagnóstico de TB en Paraguay.

## MATERIALES Y MÉTODOS

### Diseño

Se trata de una investigación operativa con diseño de cohortes retrospectivo que incluyó casos diagnosticados con TB en Paraguay de 2015-2016, a partir de la base datos del Programa Nacional de Control de Tuberculosis (PNCT).

### Contexto

Paraguay está situado en América del Sur, tiene una superficie de 406 752 km^2^ y una población de 6 725 308 habitantes (2016). El territorio nacional está dividido en dos grandes regiones (oriental y occidental) con 17 departamentos, 245 municipios y la capital, Asunción.

Posee una economía pequeña y abierta. El crecimiento económico sostenido ayudó a reducir la pobreza y a promover la prosperidad compartida. Sin embargo, la pobreza y la desigualdad de ingresos siguen siendo un reto importante. El producto ingreso bruto (PIB) per cápita en 2016 fue de 4 080,20 dólares estadounidenses (8).

El Ministerio de Salud Pública y Bienestar Social (MSPBS) financia sus actividades con recursos provenientes del Tesoro Público, no tiene separación de funciones y ejerce los roles de rectoría, financiamiento y prestación de servicios. Esta última se realiza a través de una red integrada de servicios, distribuidas en 18 regiones sanitarias que corresponden a cada uno de los departamentos y la capital (9, 10).

El PNCT depende de la Dirección General de Vigilancia de la Salud (DGVS) del MSPBS, es el ente normativo a nivel nacional y es responsable de supervisar la aplicación de la normativa, capacitar, realizar seguimiento, monitoreo y control de calidad de la información recogida desde el nivel local, además de elaborar informes sobre la situación de la TB. El diagnóstico y tratamiento de la tuberculosis está a cargo del estado y se ofrece de manera gratuita.

Existen 151 laboratorios, 12 laboratorios de cultivo, 7Xpert/TBRif^®^ y un laboratorio de referencia nacional para cultivo, tipificación y prueba de sensibilidad a drogas (PSD).

### Población y muestra de pacientes

Se incluyó todo el universo de pacientes, que corresponden a 5 141 casos con diagnóstico de TB entre enero de 2015 y diciembre de 2016. No se excluyeron datos y se tomaron las variables demográficas y clínicas que se registran de manera sistemática en el programa de tuberculosis.

### Fuente de datos y variables

La fuente fue la base de datos del PNCT. Los datos son reportados por el personal de salud a través de la anamnesis. Esta información se registra en los formularios del PNCT desde el nivel local (unidades de salud de la familia, puestos de salud, centros de salud) y se remite al nivel distrital (cabeceras distritales) que, a su vez, consolidan los datos y son remitidos al nivel regional (regiones sanitarias) que luego reportan al nivel nacional.

Los formularios de registro recogen datos sociodemográficos y clínico-epidemiológicos de casos de TB diagnosticados en servicios de salud públicos, privados y de la seguridad social. Existe una Guía de gestión de datos del PNCT que estandariza los formularios de registro con sus respectivos instructivos, además de mapas de proceso que describen los procedimientos para el registro e informe de los casos y eventos de TB. Así también, existen normas generales para asegurar la calidad de datos y metodología para aplicar controles preventivos y correctivos.

Toda información que llega al nivel nacional es validada e ingresada a una base de datos nominal en Excel^®^ en la sección de estadísticas del PNCT.

Además, la información se cruza con la base de datos del subsistema de estadísticas vitales del MSPBS, a la cual se accede de forma oficial cada tres meses. Una vez realizada la verificación, se agregan los casos que han sido reportados como fallecidos con TB consolidando e incluyendo la información en una sola (la base de datos del PNCT).

El sistema de vigilancia para la declaración de casos de TB es robusto. La implementación del programa se extiende a todas las regiones sanitarias del país. Existe un plan de capacitación que se desarrolla todos los años. Si bien se presentan casos que aparecen como fallecidos por TB en los certificados de defunción, es importante mencionar que, desde finales de 2016, Paraguay está trabajando en la capacitación de los profesionales de salud para el registro correcto de los certificados de defunción, por lo que se nota una mejora a partir de 2017 para el reporte de fallecidos. En la actualidad, se coordina en forma directa con el departamento de estadísticas vitales para discutir caso por caso, en aquellos donde el PNCT tiene dudas, de los que no han sido reportados al programa. Se ha encontrado que algunos de ellos, en efecto, no eran casos de TB.

De aquí se obtuvieron las variables relacionadas con los objetivos del estudio:

- Características sociodemográficas: edad, sexo, región sanitaria y área de residencia.
- Características clínico-epidemiológicas: inicia tratamiento, esquema de tratamiento, TDO, historia de tratamiento previo, localización anatómica, diagnóstico bacteriológico, persona viviendo con VIH (PVIH), población indígena, población privada de la libertad (PPL), contacto resistente, uso de alcohol y tabaco, DM, EPOC y personal de salud.

Se realizó la limpieza de base de datos y se estandarizó la codificación de variables. Se obtuvieron aquellas necesarias para lograr los objetivos y se corrigieron y complementaron las fechas de diagnóstico, inicio de tratamiento y egreso del programa.

### Análisis y estadísticas

Para describir las variables cualitativas, se estimaron frecuencias y porcentajes; para la edad, se estimó la mediana con los percentiles 25 y 75. Para la comparación de las variables entre las personas que egresaron del programa vivas o muertas, se usaron pruebas de chi cuadrado, riesgo relativo (RR) con un intervalo de confianza de 95%. Además, se ajustó un modelo de regresión múltiple de Poisson con errores robustos. Se usó está técnica de regresión múltiple en lugar de la regresión logística, por tratarse de un estudio de cohorte, en el que la medida de efecto adecuada es el RR y no la razón de probabilidades, que es la medida que se estima con la regresión logística (11-13).

El nivel de significación utilizado fue de 5%, se consideró que había asociación cuando el valor fue inferior a 0,05. Los datos fueron analizados mediante el *software* EpiInfo 7^®^, Stata 15^®^ y SPSS 24^®^.

### Consideraciones éticas

Para el desarrollo de la investigación se contó con autorización de la dirección del PNCT. Se obtuvo la aprobación del Comité de Ética de Investigación (CIEI), dependiente del Laboratorio Central de Salud Pública del MSPBS de Paraguay y la exoneración por parte del Comité de Revisión Ética de la OPS (PAHOERC).

Debido a la naturaleza retrospectiva y a que solo se emplearon datos ya registrados en la base de datos nacional, no fue necesario el consentimiento informado a los pacientes. La base de datos fue codificada y se omitió cualquier información que permitiera identificar a las personas.

## RESULTADOS

Durante los dos años de estudio se identificaron 5 141 pacientes con diagnóstico de TB, de los cuales 70% eran de sexo masculino. El 50% de los pacientes tenían 34 años o menos, la mayoría eran de zona urbana (61%), el tratamiento fue directamente observado en 53% de los casos, 84% eran diagnósticos nuevos y 91% eran casos de tuberculosis pulmonares.

En relación con algunas condiciones de riesgo que evalúa el programa, se encontró que 15,5% eran indígenas, PPL 11,2%, PVIH 8,4%, el 3,7% se autorreportaron como pacientes con DM y 1,7% con EPOC, 13% se reportó como contacto de un caso de TB sensible y 0,2% correspondiente a un caso de tuberculosis drogorresistente (TB-DR), el porcentaje restante no reportó tener contacto con alguien con TB.

El 66% fueron diagnosticados con bacteriología positiva (con baciloscopia, cultivo o Xpert/TBRif®), 18,6% tuvo bacteriología negativa (84% de la población fue sometida a alguna prueba de confirmación bacteriológica) mientras que 15,6% no contaban con estudios bacteriológicos y, por lo tanto, fueron diagnosticados por el cuadro clínico (cuadro 1).

Se reportaron como fallecidos 594 casos de TB, equivalente a 11,5%. De estos, 16,6% fueron pacientes que no habían sido notificados como casos de TB por los servicios de salud; sin embargo, aparecen como fallecidos por TB en el Subsistema de estadísticas vitales del MSPBS.

Se observó un mayor porcentaje de fallecidos en la población del sexo masculino, con 12,4% comparado con 9,5% en mujeres; en cuanto a la edad, se observa que las personas que fallecen son mayores de edad, con una diferencia de medianas de 16,5 años. Entre los casos que no habían iniciado tratamiento, la mortalidad fue mayor, fallecieron 43,8% (es importante aclarar que entre los estos se incluyen los casos agregados desde el Subsistema de estadísticas vitales del MSPBS), comparado con un 9,5% de personas que fallecieron entre los que sí recibieron tratamiento.

Entre los pacientes que iniciaron tratamiento (94,1%), 7% de los casos que no realizaron TDO fallecieron. En cuanto a tratamiento previo, se observó que 14,8% de fallecidos fueron con tratamiento después de la pérdida de seguimiento; seguido de aquellos que han sido clasificados como historia desconocida, con 13,4%. El 20,9% de los casos catalogados como TB sin confirmación bacteriológica, han fallecido, comparado con 8,4% de los que fueron confirmados por bacteriología. Por otra parte, la mortalidad fue mayor en personas con coinfección TB/VIH (35,9%) comparado con las personas sin la coinfección (9,3%) (cuadro 1).

Según las tasas de mortalidad ajustadas, la región sanitaria que presentó una mayor tasa de mortalidad fue Alto Paraguay (18,1 en 2015 y 23,7 en 2016), seguida de Boquerón (18,2 en 2015 y 12, 9 en 2016) y Presidente Hayes (8,7 en 2015 y 10,1 en 2016). Todas estas muertes corresponden a las regiones que se encuentran en el Chaco Paraguayo, que se caracteriza por la baja densidad poblacional y su población en su mayoría indígena. Este ajuste se realizó por el método directo, como población de ajuste se utilizó la población total a nivel país, para cada año (cuadro 2).

El análisis de regresión múltiple mostró factores de riesgo tales como: sexo masculino (RR: 1,26 IC95%: 1,1-1,50) infección con VIH (RR: 4,78; IC95%: 4,04-5,65), EPOC (RR: 1,70; IC95%: 1,19-2,42). Respecto a la edad, por cada año adicional este riesgo aumenta 3%. Como factor protector, se detectó ser persona privada de libertad (RR: 0,37; IC95%: 0,24-0,61) (cuadro 3).

## DISCUSIÓN

Este estudio es el primero que investiga la mortalidad por TB de todas las formas en todo el país, a lo largo de dos años. Como parte del estudio, se describieron características sociodemográficas, clínicas y epidemiológicas de 5 141 casos y se establecieron factores asociados a la mortalidad de los 594 reportados entre 2015 y 2016: coinfección con VIH, EPOC, sexo masculino y edad.

Entre las condiciones estudiadas para la mortalidad, se encontró que 8,4% tenía coinfección TB/VIH, 35,9% murió, en comparación con 9,3% de personas sin la coinfección, el riesgo en esta población fue 3,8 veces más alto que en las personas sin coinfección, esto es superior a la media reportada en la Región de las Américas, que asciende a 25% en 2017 (3). La reducción de la tasa de mortalidad en estos pacientes ha sido 50% menor que en personas sin VIH en los últimos tres años. De acuerdo con las estadísticas mundiales, la TB es una de las infecciones oportunistas más comunes que afectan a las PVIH y causan la mayoría de las muertes asociadas con el sida. La respuesta inmunitaria alterada conduce a una presentación clínica atípica de TB, que resulta en un diagnóstico erróneo y una progresión extremadamente rápida a la muerte (14, 15).

**CUADRO 1.** Características sociodemográficas y clínicas de personas con tuberculosis en Paraguay (2015-2016)

Características	Muerte						Valor de *P*^a^
	Sí		No		Total	
	n = 594	(%)	n = 4547	(%)	n = 5141	(%)
Sexo							
Masculino	449	12,4	1386	90,5	3610	70,2	0,002
Femenino	145	9,5	3161	87,5	1531	29,8
Edad (años) (Pc25-Pc75)	49,5 (34-66)		33 (23-51)		34 (24-53)		< 0,001^a^
Área de residencia							
Urbana	297	14,9	1684	85,1	1981	61,1	< 0,001
Rural	149	11,7	1117	88,2	1266	38,9
Inicia tratamiento							
No	133	43,8	170	56,1	303	5,8	< 0,001
Sí	461	9,5	4377	90,4	4838	94,1
Esquema de tratamiento							
Sensible	454	9,4	4345	90,5	4799	96,7	< 0,001
Resistente	4	12,5	28	87,5	32	0,6
ND	129	99,2	1	0,7	130	2,6
TDO							
No	148	6,9	1978	93,1	2126	44,6	< 0,001
Sí	250	9,9	2259	90,1	2509	52,6
ND	129	99,2	1	0,7	130	2,7
Historia de tratamiento previo							
Nuevo	489	11,2	3853	88,7	4342	84,4	0,383
Recaída	58	12,8	392	87,1	450	8,7
Tratamiento después de la pérdida de segumiento	24	14,8	138	85,2	162	3,1
Tratamiento después del fracaso	2	6,4	29	93,5	31	0,6
Historia desconocida	21	13,4	135	86,5	156	3,1
Localización anatómica							
Extrapulmonar	74	15,8	393	84,2	467	9,1	0,002
Pulmonar	520	11,1	4154	88,8	4674	90,9
Diagnóstico bacteriológico
Negativo	141	14,6	820	85,3	961	18,6	< 0,001
Positivo	287	8,4	3102	91,5	3389	65,9
Sin bacteriología	166	20,9	625	79,1	791	15,3
Persona con VIH							
No	438	9,3	4269	90,7	4707	91,5	< 0,001
Sí	156	35,9	278	64,1	434	8,4
Población indígena							
No	512	11,7	3829	88,2	4341	84,4	0,209
Sí	82	10,2	718	89,7	800	15,5
Población privada de la libertad							
No	577	12,6	3989	87,4	4566	88,8	< 0,001
Sí	17	2,9	558	97,1	575	11,1
Contacto resistente							
No	591	11,5	4539	88,4	5130	99,7	< 0,001
Sí	3	27,2	8	72,7	11	0,2
Alcoholismo							
No	563	11,4	4356	88,5	4919	95,6	0,251
Sí	31	13,9	191	86,1	222	4,3
Tabaquismo							
No	524	11,6	3988	88,4	4512	87,7	0,722
Sí	71	11,1	558	88,9	629	12,2
Diabetes mellitus							
No	572	11,5	4379	88,5	4951	96,3	< 0,001
Sí	22	11,5	168	88,5	190	3,6
EPOC							
No	571	11,3	4482	88,7	5053	98,2	< 0,001
Sí	23	26,1	65	73,9	88	1,7
Personal de salud							
No	592	11,6	4507	88,4	5099	99,1	0,167
Sí	2	4,7	40	95,3	42	0,8

^a^ Los valores *P* son de la prueba chi cuadrado excepto es de la edad, que es de la prueba U de Mann-Whitney.

VIH, virus de la inmunoedficiencia humana; ND; no disponible; EPOC, enfermedad pulmonar crónica; TDO, tratamiento directo observado.

***Fuente***: elaboración propia a partir de la base de datos del Programa de Control Nacional de Tuberculosis.

**CUADRO 2.** Tasas de mortalidad cruda y ajustada por edad según regiones sanitarias, Paraguay (2015-2016)

Región	2015			2016		
	Tasa cruda	Tasa ajustada	IC95%	Tasa cruda	Tasa ajustada	IC95%
Concepción	7,1	8,1	(4,7-13,1)	4,9	5,5	(2,8-9,8)
San Pedro	5,1	5,7	(3,5-8,8)	3,1	3,2	(1,7-5,6)
Cordillera	4,4	4,2	(2,2-7,4)	2,7	2,4	(1,1-5,1)
Gauirá	3,2	3,1	(1,2-6,6)	3,2	2,9	(1,2-6,2)
Caaguazú	3,5	3,8	(2,2-6)	2,8	2,8	(1,6-4,7)
Caazapá	2,8	3,1	0,9-7,3)	1,1	1,1	(0,1-4,5)
Itapuá	4,5	4,8	(3,1-7,1)	2,9	3,1	(1,7-4,9)
Misiones	2,5	2,5	(0,5-7,7)	5,7	5,2	(2,1-11,2)
Paraguaría	5,1	4,6	(2,14-8,2)	3,9	3,6	(1,7-6,8)
Alto Paraná	5,9	6,2	(4,4-8,6)	5,7	6,1	(4,3-8,5)
Central	3,7	3,7	(2,9-8,2)	3,5	3,5	(2,7-4,4)
Ñeembucú	2,2	1,9	(0,2-8,2)	4,5	3,3	(0,8-9,9)
Amambay	3,7	4,1	(1,5-9,07)	3,1	3,3	(1,1-7,8)
Camindeyú	4,7	5,7	(2,7-11,5)	1,3	2,9	(0,3-6,6)
Presidente Hayes	8,7	9,1	(4,4-17,1)	10,1	10,6	(5,5-18,8)
Boquerón	18,2	19,3	(9,5-35,4)	12,9	14,5	(5,8-27,5)
Alto Paraguay	18,1	19,5	(4,1-61,5)	23,7	22,9	(6,2-63,3)
Capital	5,6	5,1	(3,4-7,5)	7,9	7,2	(5,3-9,9)

IC95%, intervalo de confianza de 95%.

***Fuente:*** elaboración propia a partir de la base de datos del Programa de Control Nacional de Tuberculosis y la Proyección de Población de la Dirección General de Encuestas, Estadísticas y Censos.

**CUADRO 3.** Factores asociados a la mortalidad, casos diagnosticados en Paraguay (2015-2016)

Variables	RR	IC95%	Valor de *P*
Sexo masculino	1,26	(1,1-1,5)	0,006
Edad en años	1,03	(1,022-1,034)	< 0,001
Localización extrapulmonar	1,03	(0,84-1,28)	0,726
Paciente previamente tratado	1,17	(0,96-1,42)	0,102
Persona con VIH	4,78	(4,04-5,65)	< 0,001
Población indígena	1,11	(0,89-1,37)	0,339
Población privada de la libertad	0,37	(0,24-0,61)	< 0,001
EPOC	1,7	(1,19-2,42)	0,003
Personal de salud	0,46	(0,12-11,82)	0,197

RR, riesgo relativo; IC95%, intervalo de confianza de 95%; VIH, virus de inmunodeficiencia humana; EPOC, enfermedad pulmonar obstructiva crónica.

***Fuente:*** elaboración propia a partir de la base de datos del Programa de Control Nacional de Tuberculosis.

En este sentido, se hace necesaria la efectiva implementación de las actividades colaborativas TB/VIH, con énfasis en la detección sistemática de síntomas de tuberculosis en PVIH. En Paraguay existen 13 servicios de atención integral (SAI) que atienden PVIH, distribuidos en diferentes regiones sanitarias, lo que constituye una limitación para el acceso de pacientes que residen en las demás regiones. Además, la mayoría de los casos (casi 70%) acuden hasta la capital para ser atendidos y retirar sus medicamentos antirretrovirales todos los meses.

En cuanto al diagnóstico, si bien el país dispone de pruebas rápidas de biología molecular y en las normas nacionales se establece como grupo prioritario a PVIH, es necesario asegurar suficientes cartuchos para la solicitud de la prueba y agilizar el diagnóstico en esta población, lo que tendrá un impacto en la reducción de la mortalidad (16, 17).

La EPOC resultó ser otro factor importante para la mortalidad, dado que aumenta el riesgo en 70%. En el 2016 fallecieron tres millones de personas por EPOC, que es la tercera causa de mortalidad en el mundo (18). En algunos estudios, se demostró que tienen un riesgo tres veces mayor de desarrollar TB activa en comparación con la población general. Además, el riesgo de muerte aumenta dos veces más, en el primer año después del diagnóstico de TB, en comparación con los sujetos de control de la población general que tienen tuberculosis (19).

El aumento de la carga de EPOC es un motivo de preocupación. El deterioro del sistema inmunitario innato y la terapia con corticosteroides en pacientes con EPOC pueden aumentar el riesgo de manifestación de TB. Del mismo modo, el daño pulmonar causado por la TB puede aumentar la susceptibilidad al desarrollo posterior de EPOC, incluso después de completar el tratamiento antituberculoso efectivo (20, 21).

A medida que aumenta la edad se incrementa el riesgo de fallecer por TB, esto puede deberse a comorbilidades y al declive natural de la inmunidad, la respuesta inmunitaria se va deteriorando y esto contribuye a la reactivación de la TB latente, lo que hace más propensos a desarrollar la enfermedad. Esto, sumado al diagnóstico tardío, aumenta la posibilidad de un desenlace fatal (22, 23).

Se diagnostica más tuberculosis en hombres que en mujeres y, también, son quienes más mueren. Se ha demostrado que en países de renta más baja se registra el doble de casos de TB en los varones, esto se suele atribuir a características biológicas y epidemiológicas, así como a obstáculos socioeconómicos y culturales para el acceso salud. También lo refieren estudios de África y Europa (24-27).

Otra información relevante es que, de 94% de los casos que iniciaron tratamiento, 44,6% no ha realizado TDO. Con base en los registros, casi la mitad de los pacientes no estuvo adherida a ninguna modalidad de TDO. Este hecho debe considerarse, pues podría incidir en la mortalidad por TB en un futuro, como describe la literatura.

Existen dificultades para el TDO, sobre todo en los hospitales especializados y la seguridad social y zonas distantes. Para afrontar algunas dificultades, en comunidades muy lejanas, como la región occidental (Chaco paraguayo) aún se busca comprometer a líderes comunitarios para la implementación del TDO.

Es de destacar también que 15,3% de los pacientes fueron diagnosticados por clínica (frotis negativo o métodos diagnósticos accesorios, etc.), de los cuales falleció 20,9%. Una revisión sistemática asocia la falta de confirmación bacteriológica a un riesgo mayor de muerte (5). Algunos estudios señalan que esto puede deberse a que no se identifican de manera oportuna los factores de riesgo y los signos de alarma, el diagnóstico es tardío, o que sean personas inmunocomprometidas (p. ej., con VIH) en quienes no se puede confirmar por bacteriología (14, 16, 28).

Sin embargo, representó un factor de protección que la población sea privada de libertad, donde se reportó 2,9% de fallecidos. Esto es contrario a lo que se describe en la literatura, donde la mortalidad en PPL es mayor. Señalan que factores tales como el hacinamiento y la propagación de la enfermedad, más la mala adherencia durante y después del tratamiento (sobre todo por la pérdida de seguimiento luego de salir de prisión) representa un riesgo de muerte (29, 30).

En concordancia con los reportes nacionales, se evidenció un mayor éxito de tratamiento en PPL (80% en 2016) en comparación con la población general (65% para el mismo año). Esto se debe a que Paraguay ha intensificado las estrategias en prisiones, implementando los programas de TB en los centros penitenciarios, donde se garantiza el TDO.

A diferencia de otros artículos, la condición de previamente tratados no se demostró como factor asociado a la mortalidad, aunque estos hayan representado 12,4% de los casos, con un porcentaje de fallecidos del 34%. De acuerdo con la historia de tratamiento previo, 14,8% fueron pacientes perdidos en el seguimiento. Es importante señalar que, con el cruce de datos que realiza en PNCT con el Subsistema de estadísticas vitales, se buscan los casos de TB, incluso los previamente tratados y se agregan a la base de datos del PNCT (31).

Este estudio tiene algunas limitaciones, entre ellas que en los datos del programa de TB no se registran datos suficientes acerca del tratamiento antirretroviral ni el recuento de CD4 de los pacientes coinfectados, ya que esa información lo maneja el programa de VIH. Hasta 2016, la base de datos nominal del PNCT tenía la limitación de que no se distinguía en una sola variable, la fecha de diagnóstico. Para obtener este dato, se debía recurrir a otras variables, dependiendo si el caso tenía confirmación bacteriológica o diagnóstico clínico, por lo que no se pudo hacer el cálculo del tiempo transcurrido entre la fecha de diagnóstico y fecha de fallecimiento.

Las fortalezas de este estudio incluyen, en primer lugar, el tamaño de muestra, que abarca todos los casos diagnosticados con TB y todos los fallecidos con la enfermedad en Paraguay, con una base de datos que ha permitido obtener información sociodemográfica, clínica y epidemiológica. El cruce de datos entre la base de datos del PNCT y la del Subsistema de estadísticas vitales permite la calidad de la información en cuanto a la mortalidad por TB en el país.

Otra fortaleza es la estimación de la medida adecuada para el diseño del estudio; este es un estudio de cohorte y la medida de riesgo adecuada es el riesgo relativo. Es frecuente encontrar en la literatura médica estimación de razón de probabilidades en estudio de cohorte o en estudios transversales; esto no es adecuado, porque la razón de probabilidades sobreestima el RR y la razón de prevalencia y solo hace una buena aproximación si la frecuencia del evento estudiado es menor a 10%. Diferentes autores han demostrado que la regresión de Poisson hace una buena estimación de los riesgos relativos (12, 32, 33).

## CONCLUSIÓN

El mayor riesgo de muerte lo presentan los hombres y las personas con coinfección TB/VIH y el menor riesgo, las personas privadas de la libertad.

Es necesario mejorar el diagnóstico y seguimiento a los casos de TB, con la efectiva implementación del tratamiento directamente observado (TDO) así como el manejo oportuno de enfermedades asociadas como VIH y EPOC.

## Contribuciones de las autoras

AME contribuyó a la idea del tema a ser investigado, elaboración del protocolo, gestión y limpieza de la base de datos, interpretación de resultados, redacción de todos los borradores del manuscrito. LL contribuyó al diseño, análisis de datos, interpretación, corredacción del manuscrito y revisión de todas las versiones y la asistencia técnica. CM colaboró en la revisión de documentos, revisión bibliográfica, ajuste de protocolo y diferentes versiones del manuscrito. SA participó en la revisión del protocolo y el manuscrito, y autorización para el acceso a la base de datos del PNCT. EA participó en el diseño del estudio y la revisión de protocolo, además de asistencia técnica, revisión y lectura del primer borrador hasta la redacción del artículo. Todas las autoras han leído y aprobado el manuscrito final para su presentación.

## Agradecimientos

Esta investigación se llevó a cabo mediante la Iniciativa de Capacitación Estructurada en Investigación Operativa (SORT IT, por sus siglas en inglés), una alianza mundial dirigida por el Programa Especial de Investigación y Capacitación de Enfermedades Tropicales de la Organización Mundial de la Salud (OMS/TDR) y el Departamento de Enfermedades Transmisibles y Determinantes Ambientales de la Salud de la Organización Panamericana de la Salud (OPS).

## Financiamiento

Se obtuvo financiamiento de la oficina subregional Andina de la OPS. Los financiadores no desempeñaron ningún papel en el diseño del estudio, la recopilación y análisis de los datos, la decisión de publicar ni la elaboración del artículo.

## Declaración

Las opiniones expresadas en este manuscrito son únicamente responsabilidad de los autores y no reflejan necesariamente los de la *Revista Panamericana de Salud Pública* o la Organización Panamericana de la Salud.
